# Open Preperitoneal Inguinal Hernia Repair, TREPP Versus TIPP in a Randomized Clinical Trial

**DOI:** 10.1097/SLA.0000000000005130

**Published:** 2021-08-02

**Authors:** Willem J. V. Bökkerink, Giel G. Koning, Patrick W. H. E. Vriens, Roland M. H. G. Mollen, Mitchell J. R. Harker, Robin K. Noordhof, Willem L. Akkersdijk, Cees J. H. M. van Laarhoven

**Affiliations:** ∗ Department of Surgery, Radboud University Medical Centre, Nijmegen, the Netherlands; † Department of Surgery, ZGT Hospital Almelo, Almelo, the Netherlands; ‡ Department of Surgery, Elisabeth Tweesteden Hospital, Tilburg, the Netherlands; § Department of Surgery, Gelderse Vallei Hospital, Ede, the Netherlands; ¶ Department of Surgery, St Jansdal Hospital, Harderwijk, the Netherlands.

**Keywords:** chronic pain, CPIP, inguinal hernia, mesh, open, pain, posterior, preperitoneal, TIPP, TREPP

## Abstract

The aim of this study was to compare chronic postoperative inguinal pain (CPIP) in patients with an inguinal hernia after the TransREctus Sheath PrePeritoneal (TREPP) and the TransInguinal PrePeritoneal Technique (TIPP).

**Background:**

The preperitoneal mesh position for inguinal hernia repair showed beneficial results regarding CPIP with low recurrence rates. Two open preperitoneal techniques, TREPP and TIPP, were compared in a randomized clinical trial with the hypothesis of fewer patients with CPIP after TREPP due to complete avoidance of nerve contact.

**Methods:**

Adult patients with a primary unilateral inguinal hernia were randomized to either TREPP or TIPP in four hospitals. Before the trial's start the study protocol was ethically approved and published. Outcomes included CPIP after 1 year (primary outcome) and recurrence rates, adverse events, and health-related quality of life (secondary outcomes). Follow-up was performed at 2 weeks, 6 months, and 1 year.

**Results:**

Baseline characteristics were comparable in both groups. Pain was less often present after TREPP at 2 weeks and 6 months, but CPIP at rest at 1 year was comparable: 1.9% after TREPP vs 1.4% after TIPP, *P* = 0.535). The overall recurrence rate was higher in the TREPP group, 8.9% vs 4.6%, *P* = 0.022). Corrected for a learning curve for TREPP, no significant difference could be assessed (TREPP 5.7% and TIPP 4.8%, *P* = 0.591).

**Conclusion:**

Both the TREPP and TIPP technique resulted in a low incidence of CPIP after 1-year follow-up. The TREPP method can be considered a solid method for inguinal hernia repair if expertise is present. The learning curve of the TREPP techniques needs further evaluation.

**Trial Registration:**

ISRCTN18591339

Chronic postoperative inguinal pain (CPIP) is an important complication after inguinal hernia repair. Factors like nerve damage and mesh position might be associated.^1,2^ In the endoscopic techniques such as the totally extraperitoneal procedure (TEP) and TransAbdominal PrePeritoneal (TAPP), the preperitoneal mesh placement resulted in decreased CPIP rates (<5%).^1^ Endoscopic techniques have the disadvantages of extensive learning curves, higher costs, need for general anesthesia and a risk of major adverse events.^1,3^ An open preperitoneal alternative, the TransInguinal PrePeritoneal procedure (TIPP) showed favorable results in clinical trials^4^ such as the TULIP trial^5^ (same research group as for the present study). The TULIP results^5^ lay base for the present ENTREPPMENT trial^6^ in which the TransREctus sheath Preperitoneal (TREPP) technique^7^ was compared to the TIPP method. The TREPP technique was developed to further reduce CPIP by avoiding dissection of the inguinal canal. Other TREPP principles are: an open technique with a non-fixated preperitoneal mesh based on the biomechanical upstream principle.^8^ Previous retrospective studies were promising^8–12^ and contributed to the hypothesis of the ENTREPPMENT trial that less patients would suffer from CPIP after TREPP compared to TIPP.

## METHODS

### Study Design and Participants

The ENTREPPMENT trial was a prospective, multicenter, single-blinded, clinical trial randomly assigning 800 patients to either a TREPP or a TIPP operation. The trial sequels the TULIP trial (TIPP superior to Lichtenstein).^5^ The 1:1 web-based block-randomization process, stratified for center, was performed for each patient just before treatment by the surgeon. All recommendations, including those on allocations and blinding of the Cochrane Handbook, were followed.^13^ The trial's protocol was registered (ISRCTN 18591339) and published before the start of inclusion.^6^ The Medical Research Ethics Committee Arnhem-Nijmegen approved the study (file number 2012/060).

Patients were recruited in 4 Dutch hospitals (1 academic, 3 nonacademic in respectively Nijmegen, Tilburg, Ede, and Harderwijk). All male and female patients with a primary, unilateral, clinically evident, inguinal hernia, 18 to 80 years of age, were invited to participate in the trial if the American Society of Anesthesiologists (ASA) classification was ≤III.^6^ Exclusion criteria were: recurrent, femoral, (large) scrotal or incarcerated hernias, psychiatric disease or language barriers making follow-up unreliable and previous preperitoneal groin events. All included patients provided written informed consent.

### Surgical Techniques

The techniques have previously been described.^7,14^ TREPP in short: the preperitoneal space is entered by opening the anterior rectus sheath, retracting the rectus muscle and epigastric vessels medially and opening the transverse fascia. With gentle movements of a finger the preperitoneal space is developed both medially (Retzius’ space) and laterally (Bogros’ space). The peritoneal sac is retracted cranially for an overview of the preperitoneal space to reduce a lateral, medial or femoral hernia. A mesh is inserted in preperitoneal position covering the complete myopectineum of Fruchaud. No fixation is necessary. The rectus sheath, Scarpa's fascia, and skin are closed.

The TIPP technique was performed according to the TULIP trial.^5,14^ Using the transinguinal approach the hernia is identified. Inguinal nerves are identified and spared. The hernia sac is reduced, not resected. The hernia orifice is used as an entrance to the preperitoneal space and again developed by a finger to safely place a mesh in the preperitoneal space covering the lateral, medial and femoral hernia sites. No mesh fixation is needed and a standard layered closure is performed.

A Polysoft 16.0 by 9.5 cm low-weight polypropylene mesh with recoil ring (C.R. Bard part of BD Interventional, Vianen, The Netherlands) was used in both the TREPP and TIPP techniques. Spinal anesthesia was preferred, though general anesthesia was not excluded. All periprocedural protocols were standardized, including wound infiltration with 10 mL ropivacaine 7.5 mg/mL and postoperative analgesic treatment (paracetamol 1000 mg 4 times daily, naproxen 250 mg 3 times daily, oxynorm 5 mg 6 times daily, all when needed). The 1 to 2 cm difference in incision location between the 2 techniques was considered noticeable for surgical professionals only. All patients were instructed to restart daily activities, including work, as soon as the pain would allow them to.

The TREPP and TIPP techniques were performed by dedicated surgeons and supervised residents. Before the trial all surgeons participated in group sessions in the operation theater for uniformity in each step of both techniques. Hernia surgeons who were not skilled in one or both of the techniques received training from experts before their participation to the trial. Based on experts’ opinions, a minimum of 10 supervised procedures per technique were recommended in this training phase.^15^

### Endpoints

Primary outcome was defined as the number of patients with CPIP at 1 year postoperatively (CPIP was defined as any pain persisting at least 3 months postoperatively).^16^ For more clinical relevance and to promote comparison with international literature,^1,2^ more details on the outcome of pain are reported. Pain was measured at rest and during activity only, both dichotomous and with the Visual Analogue Scale (VAS) for pain, VAS_Pain._ The terms “rest” and “activity” were used to increase generalizability among patients with different physical abilities. Secondary outcomes included surgical site infections,^17^ clinically evident recurrent hernia, sensory disturbances (measured with the pin-prick test), and reoperations. Further outcomes were length of hospital stay, operative time, return to daily activities including work, and Health Status (or: health-related “quality of life"). Patients visited the outpatient departments preoperatively, 2 weeks, 6 months, and 1 year postoperatively for an interview, physical examination and Health Status questionnaires.^6^ The first two weeks patients filled in a Pain Diary including VAS_Pain_ and the Pain Disability Index (PDI).^18^ The outcome observer was unblinded by definition but allocation concealment for the patient was maintained until the last follow-up check was finished.

### Data Storage

Data were recorded with paper Case Report Forms (CRFs) by the patients and medical professionals. Data were stored on site during inclusion and follow-up phases after which it was digitalized (Electronic Data Capture system, Castor EDC, Amsterdam, the Netherlands). The CRFs are stored in the storage facilities of the Radboud University Medical Center (UTS Verkroost, Nijmegen, boxes R103-112). Only the principle investigator and study coordinator can access the data. The external party funding part of the trial had no access to the data.

### Statistics and Reporting

The power calculation and statistical plan were published in the trial protocol. In short, an absolute, overall (continuous plus activity-related) CPIP reduction from 12% after TIPP^5^ to 6% after TREPP was hypothesized. Eight-hundred patients were required, based on an 80% power, 0.05 two-sided alfa and a 10% anticipated loss to follow-up. Intention-to-treat analyses were performed. Statistical significance was tested by means of the *χ*^2^ test or Fisher exact test (in case of numbers <5) for dichotomous data and the independent *t* test (normally distributed) or Mann–Whitney test (skewed data) for continuous data. A 2-sided *P* value of <0.05 was considered significant and 95% confidence intervals were calculated. Repeated measurements were analyzed with a mixed model and Likelihood ratio test. Imputation was not performed. Calculations were done with SPSS version 25 (2017, SPSS Inc, Chicago, IL) and SAS version 9.4 (SAS Institute, Cary, NC). This report follows the Consolidated Standards of Reporting Trials (CONSORT) statement.^19^ The funder had no role in the trial's design, data collection, data analysis, result interpretation, and in outcome reporting.

## RESULTS

**FIGURE 1:**
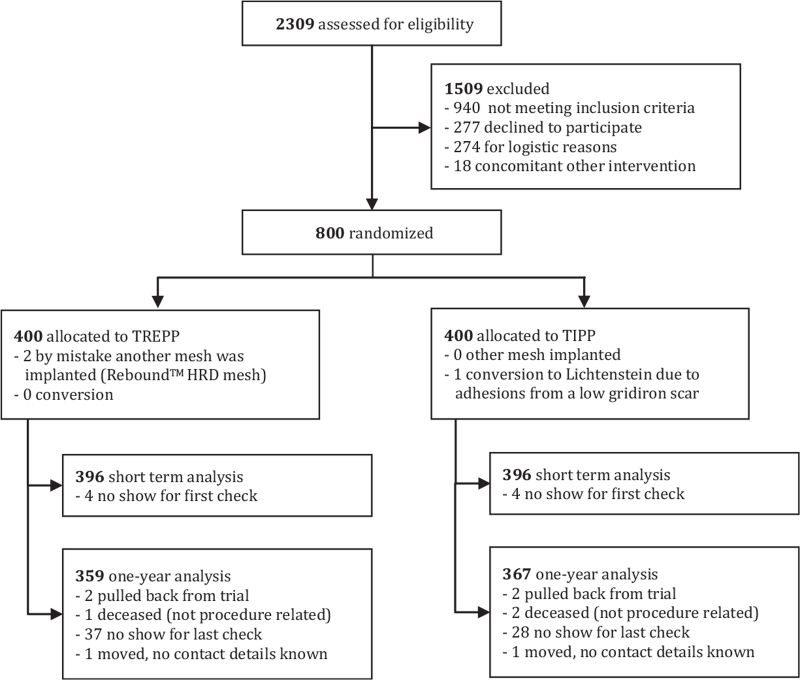
Flowchart screening, randomization and follow-up.

Between February 2014 and February 2017, eight-hundred patients were randomly allocated to the TREPP (n = 400) and TIPP (n = 400) techniques. Short-term analyses could be performed on data from 99% of the randomized patients, the 1-year analyses in 91% (Fig. 1).

### Baseline Characteristics

Table 1 shows that the patient characteristics were comparable among the groups, confirming adequate randomization. Furthermore, no significant differences were found in medical history, smoking, use of medication, or history of pain complaints.

**TABLE 1:** Baseline Characteristics

	TREPP (*n* = 396)	TIPP (*n* = 396)
Sex, % male	98.2	98.0
Age, y, mean (SD)	57.9 (13.9)	57.5 (13.4)
BMI, mean kg/m^2^ (SD)	25.1 (2.8)	25.1 (2.9)
Side of hernia		
Left (%)	189 (47.7)	172 (43.4)
Right (%)	207 (52.3)	224 (56.6)
ASA fitness grade		
I (%)	221 (55.8)	221 (55.8)
II (%)	164 (41.4)	164 (41.4)
III (%)	11 (2.8)	11 (2.8)
Preoperative complaints		
None (%)	33 (8.3)	29 (7.3)
Discomfort (%)	108 (27.3)	113 (28.5)
Pain	254 (64.2)	254 (64.1)
At rest, median VAS (IQR)^∗^	23 (13.5–31.75)	22 (10–31)
With activity only, median VAS (IQR)^∗^	35 (21–58)	37.5 (22–56)
Unknown (%)	1 (0.3)	0
Employment type		
Paid job (%)	228 (57.6)	236 (59.6)
Voluntary work (%)	26 (6.6)	25 (6.3)
Retired or unemployed (%)	140 (35.4)	132 (33.3)
Unknown (%)	2 (0.5)	3 (0.8)

BMI indicates body mass index; IQR, 25%–75% interquartile range; SD, standard deviation; VAS, visual analogue scale.

∗ Only the VAS of pain patients are presented.

Hernia-specific characteristics were comparable. Inguinal hernias were symptomatic in 92.2% of the patients. During TREPP surgery, inguinal hernias were lateral in 253 (63.9%), medial in 123 (31.1%), or combined (pantaloon hernia) in 18 (4.5%), versus 250 (63.1%) lateral, 121 (30.6%) medial, and 22 (5.6%) combined hernias found during TIPP surgery. The EHS-classification scores were comparable. During respectively TREPP and TIPP surgery, 1 versus 2 patients appeared to have a femoral hernia that was preoperatively considered an inguinal hernia based upon physical examination.

### Perioperative Results

**FIGURE 2:**
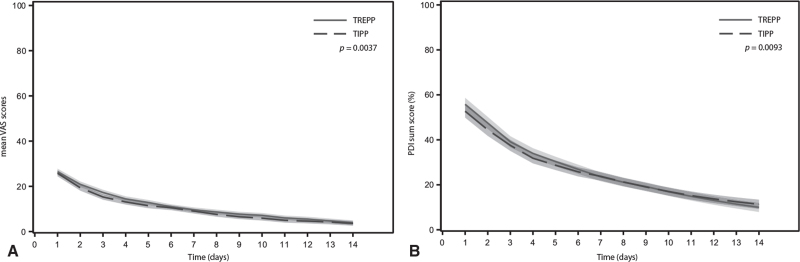
Two week recovery trends of (A) mean VAS scores and (B) Pain Disability Index sum scores. Bands represent 95% confidence intervals.

Table 2 shows the main perioperative results. Surgery was mostly performed in day care under spinal anesthesia. Non relevant small differences in median operation duration (23 vs 25 minutes, *P* = 0.002) and incision length (50 vs 52 mm, *P* = 0.001) were found when comparing TREPP with TIPP operations. Supervised residents performed more TIPP than TREPP operations (10.9% vs 5.8%, *P* = 0.010). Minor complications, not critical for decision making according to the Grading of Recommendations Assessment, Development and Evaluation (GRADE) Working group^3,20^ did not differ between the groups (eg, infections, wound dehiscence, or urine retention). Three patients with a bleeding (once after TREPP and twice after TIPP) needed surgical treatment. Another patient required surgical drainage of a wound abscess under general anesthesia (after TREPP, mesh not involved). All these patients recovered fully. No differences were found in VAS_Pain_ scores of the first hours postoperatively (mixed model, Likelihood ratio test). In both groups the median time to return to daily activity was 6 days. Pain and limitations in daily activities were reported in a two-week diary (Fig. 2). The presence of any pain (both at rest and during activity only) at 2 weeks and 6 months follow-up differed significantly: respectively 31.8% of TREPP versus 39.8% of TIPP patients (*P* = 0.025) and 10.8% (TREPP) versus 17.7% (TIPP) (*P* = 0.023).

**TABLE 2:** Two-Week Outcomes

	TREPP (396)	TIPP (396)	*P*
Duration of operation, min, median (IQR)	23 (18–29)	25 (20–31)	0.002^∗^
Spinal (%)	236 (59.6)	240 (60.6)	0.772
Incision length, mm, median (IQR)	50 (47–55)	52 (49–60)	0.002^∗^
Day care (%)	386 (97.5)	387 (97.7)	0.816
Performed by supervised resident (%)	23 (5.8)	43 (10.9)	0.010
Complications			
Surgical site infection^†^ (%)	14 (3.6)	17 (4.3)	0.592
Wound dehiscence (%)	2 (0.5)	7 (1.8)	0.095
Urine retention (%)	4 (1.0)	2 (0.5)	0.409
Post spinal headache needing intervention (%)	1 (0.3)	2 (0.5)	0.566
Early recurrence, <2 wk (%)	7 (1.8)	0	0.008
Re-surgery needing anesthesia (%)	4 (1.0)	2 (0.5)	0.412
Early recurrence	2	0	
Bleeding	1	2	
Infection/abscess^‡^	1	0	
Return to daily activities, median days (IQR)	6 (3–9)	6 (3–10.75)	0.434

IQR indicates 25%–75% interquartile range.

∗ Not normally distributed, so Mann–Whitney *U* test.

† All surgical site infections were superficial according to the Center for Disease Control and Prevention definition (ref).

‡ In this case the mesh did not have to be removed; a supra-fascial abscess was surgically drained under general anesthesia.

### One-Year Outcomes

The incidence of any CPIP (including pain during activity only) 1 year postoperatively was 7.2% and 7.9% after TREPP and TIPP. More in detail, CPIP at rest 1 year postoperatively was present in 12 patients, 7 after TREPP (1.9%) versus 5 patients after TIPP (1.4%) (*P* = 0.535). These numbers precluded multivariate regression analyses to explore potential risk factors. Treatment for this pain was not wished for by 3 versus 1 of these patients since symptoms were mild. Furthermore, pain only present during activity was reported by 43 patients (TREPP *n* = 19, 5.3% vs TIPP *n* = 24, 6.5%, *P* = 0.477). In 7 of these 43 patients the mesh was palpated (lateral point) and painful during activity.

Fewer patients noticed groin numbness after TREPP compared to TIPP (Table 3), but this could not be confirmed with the more objective pin-prick test. Sensory disturbances after respectively TREPP and TIPP (pin-prick test) were present in the dermatome of the Iliohypogastric nerve in 30 and 29 patients, the Ilioinguinal nerve in 32 and 48 patients and the genital branch of the Genitofemoral nerve in 2 and 5 patients (all *P* > 0.05). Health Status parameters from the SF-36 questionnaire showed no clinically relevant differences between the groups (Supplemental Digital Content).

**TABLE 3:** One-Year Outcomes

	TREPP (*n* = 359)	TIPP (*n* = 367)	*P*
Chronic postoperative inguinal pain			
Patients with continuous CPIP (%)	7 (1.9)	5 (1.4)	0.535
VAS at rest, median (IQR)^∗^	31 (14–80)	22 (13–55)	0.755^∗∗^
VAS activity, median (IQR)^∗^	49 (40–80)	60 (35.5–71.5)	1.000^∗∗^
Pain Disability Score, median (IQR)^∗^	21.5 (12–29)	35 (N/P)	0.381^∗∗^
Patients with activity related CPIP (%)	19 (5.3)	24 (6.5)	0.477
VAS activity, median (IQR)^∗^	30 (19–54)	24 (19.25–46.25)	0.874^∗∗^
Pain Disability Index, median (IQR)^∗^	4 (0–10)	7 (1–11)	0.537^∗∗^
Sensory disturbance noticed by patient (%)	23/352 (6.5)	40/365 (11.0)	0.036
Sensory disturbances at pin-prick test (%)	51/355 (14.4)	68/363 (18.7)	0.116
Patients with groin hyperesthesia	0/51 (0%)	7/67 (10.4%)	0.019
Periejaculation pain, male only (%)	2/343 (0.6%)	3/356 (0.8%)	1.000
Recurrences (%)	32 (8.9)	17 (4.6)	0.022
Subgroup: leaving out every surgeon's first 10 TREPP patients	16/250 (6.4)	17/367 (4.6)	0.338
Re-surgery needing anesthesia (%)	30 (8.4)	16 (4.4)	0.027
For recurrence	24	10	
For CPIP	3	4	
For bleeding or abscess	3	2	

IQR indicates 25%–75% interquartile range; N/P, not possible due to low numbers.

∗ Scores presented only concern the patients reporting pain.

∗∗ Not normally distributed, Mann–Whitney *U* test; the PDI score is displayed as absolute total numbers, possible range 0 to 70 based on 7 items from 0 to 10.

The recurrence rates displayed in table 3 show more recurrent hernias after TREPP compared to the TIPP technique (8.9% vs 4.6%, *P* = 0.011). TREPP recurrences occurred often early in the postoperative phase. This hypothesized a learning curve effect. When taken experience into account, no statistically significant difference was found anymore (6.4% vs 4.6%, *P* = 0.338). If more expertise was present, recurrence rates were low: a TIPP operation by surgeon with more TIPP experience (8/14 participating surgeons) resulted in 3.6% of cases in recurrent hernia. For the TREPP operations by surgeons with more TREPP experience (4/14) this was 2.1%. Eleven of the 49 patients with a recurrence had no or minimal symptoms and abstained surgical treatment. The other recurrences were treated as follows: 7 re-TREPPs, 4 re-TIPPs, 21 Lichtenstein's (17 after TREPP, 4 after TIPP), 2 TAPPs (after TIPP), and 4 were awaiting their treatment.

## DISCUSSION

The ENTREPPMENT trial tested the hypothesis of less CPIP after TREPP versus TIPP by randomizing 800 patients. Although open surgery with posterior route for preperitoneal mesh placement is not new,^21^ the simply described TREPP method^7^ has gained popularity because it theoretically fulfills all Reinpold's recommendations.^22^ This trial reports a short operation time, quick recovery, and low complications rates after both techniques. The primary endpoint of continuous CPIP was both low and not significantly different after TREPP versus TIPP operations. Two-week and 6-month postoperative pain and persisting numbness were more often present after TIPP. An unanticipated learning curve effect could explain the high recurrence rate after TREPP.

With a 1b level of evidence (LoE),^23^ this trial is the first to confirm the earlier published promising results after TREPP^8–12^ and good results after TIPP surgery.^4,5^ Retrospective TREPP studies report CPIP rates of 1.7%^10^ and 5.3%^9^ (LoE 3b-4) and recurrence rates of 1.2%,^9^ 1.7%^10^ and 3.9%^11^ (LoE 3b-4) with significant follow-up variation (2 weeks–2 years). The more extensive evidence on TIPP surgery, mainly compared to Lichtenstein's technique, shows <5% CPIP rates (LoE 1b–2b),^4,5,24–26^ ∼3% recurrence rates (LoE 1b-2b),^4,5,24,25,27^ and quicker recovery.^5^ Some studies found lower short-term pain scores after TIPP when compared to Lichtenstein's (LoE 1b–2b)^25,27^ and good long-term results were reported (LoE 1b).^28^ Some data show a lower percentage persisting numbness after preperitoneal repair (TREPP 15.3% LoE 4^9^, TIPP 10.6% LoE 1b^5^, TEP 4.3%^29^) compared to Lichtenstein (12.5%–51.0% LoE 1a-b^3,5^); however, data are scarce.

Literature comparison with these ENTREPPMENT trial's results is troublesome due to: TIPP technique modifications,^4^ different pain definitions or measurement (reporting),^1,2,30^ and flawed follow-up.^4^ Nevertheless, the clinically relevant pain incidences after both TREPP and TIPP in this trial are considered low. Secondary outcomes are within range, apart from the high recurrence rates. When correcting for surgeon's experience or potential learning curve, these rates return to 6.4% and 4.6%, the upper limits of reported ranges in literature.^1^ A similar effect of surgeon's experience is known from the TEP technique, for example, reported in a large TEP versus Lichtenstein trial (LoE 1b)^29^ where unexperienced versus experienced surgeons had respectively 8.0% and 1.9% recurrences after TEP.

The hypothesis in this trial of less chronic pain due to avoidance of intraoperative nerve damage is not confirmed. Although literature associates neuropathy with CPIP,^2^ the role of other influencers like mesh type, inflammatory response, and fibrotic processes is complex and yet unknown. Although less neuropathy after preperitoneal techniques is reported, this problem is not fully resolved and could be partially explained by the “naked nerves” in the parietal compartment of the preperitoneal space.^31^ The CPIP (at rest) incidence after TIPP in the ENTREPPMENT trial (1.4%) was lower compared to the TULIP trial (3.5%).^5^ Methodology was similar, but the multicenter ENTREPPMENT trial was later in time. Increased experience and continuing awareness to the problem of CPIP by the whole research group might have positively influenced this reduction.

After these open preperitoneal techniques more lateral than medial recurrences were diagnosed. This contrasts with recurrence patterns in the Lichtenstein's and endoscopic procedures.^32^ It could be hypothesized that Bogros’ space is digitally more difficult to dissect than Retzius’ space. The interruption in the Polysoft mesh’ recoil ring might also contribute due to mesh folding laterally. More importantly, there seems a learning curve effect for recurrences, particularly for the TREPP technique. Further learning curve studies will provide answer to see which minimal number of supervised procedures should be advised and what can be done to minimize harm by learning surgeons.

An effort was made to reduce risks of bias in this trial. With this approach and due to the multicentric, randomized nature, a higher level of evidence can be obtained. Unfortunately, the unanticipated learning curve effect for recurrences requires that interpretation should be cautiously undertaken. Furthermore, in this trial no comparison was made to the Lichtenstein technique that is historically more often used as reference technique. However, the Lichtenstein technique is not the criterion standard anymore in the guidelines^1^ and may disappear as first choice treatment of primary inguinal hernia repair. A comparison to the endoscopic preperitoneal techniques seems therefore an interesting next step, together with a long-term evaluation of the present population and an exploration of the potential benefit of open preperitoneal repair methods in low- and middle-income countries.

In conclusion, this randomized comparison of TREPP versus TIPP showed low and equal rates of chronic pain. Some differences were found and favor TIPP for learning curve associated recurrences, whereas TREPP is favored for better short-term pain outcomes. No explicit advantage of either technique could be drawn from this trial.

## ACKNOWLEDGMENTS

The authors thank the following ENTREPPMENT trial facilitators: all participating patients, contributing nurses, outpatient department staff, and junior doctors of the 4 recruiting hospitals. Furthermore by name, we thank drs. J. van Dijk, drs. F. Boekhoudt, drs. C. van der Waal, dr. C.S. Andeweg, drs. M.W.A. van Tilburg, dr. O.R. Buyne, dr. V. Leferink, drs. T. van Egmond, dr. M.S. Ibelings, drs. W.R. Spanjersberg. The authors thank dr. A. de Haan for his statistical support, and Mr. B. Krijnen, Mrs. T. Tromp, and Mrs. M. Abdullah for their aid in data entry.

## Supplementary Material

Supplemental Digital Content

## DISCUSSANTS

### Francesco Corcione (Napoli, Italy)

I would like to thank the authors for this interesting study, and the ESA for the honor of being the first discussant of this article. The authors evaluated 2 procedures adopting a preperitoneal approach in patients with a primary unilateral inguinal hernia. This route is usually used to treat recurrent hernia following an anterior approach. This type of hernia is excluded in the trial.

I have the following comments and questions. First, the most recent international guidelines suggest treating recurrent hernia with an endoscopic approach. Have you hypothesized a future comparison in this setting with the endoscopic approach?

Second, the authors concluded their article with the sentence: “TREPP method can be considered a solid method for inguinal hernia repair if expertise is present.” However, the authors report a recurrence rate for TREPP of 6.4% just at 1 year, even when we correct for the surgeon's experience or a potential learning curve. Actually, the recurrence rate for the Lichtenstein or endoscopic approach is about 1% to 2%. How do you explain that this is higher than the aforementioned?

Finally, concerning a tailored approach for inguinal hernia repair, what is the most appropriate indication for these open preperitoneal techniques?

### Response From Willem J.V. Bökkerink (Nijmegen, The Netherlands)

In answer to your first question, our present focus is on the open techniques, since 50% of the procedures are still performed in an open fashion in our country and in many other countries around us. We agree, however, that a comparison with the endoscopic approach is an interesting and logical next step. A study like this should not only evaluate the complications, but also the learning curve and cost effectiveness.

Regarding your second question, we fully agree that the recurrence rate is too high. I have a few remarks regarding this. First, correcting for the surgeon's experience was only a first, rough correction. It does indicate that there is the presence of a learning curve effect, but we do not know to what extent this effect is present. Secondly, the recurrence rates of the previous trials and retrospective studies were lower, which perhaps suggests that the true recurrence rate might be different from the current numbers. Finally, you take a number of 1% to 2% as a reference number for recurrences; however, we consider this to be an underestimation, especially when we take a longer follow-up time, as the guidelines recommend, into account.

Finally, to answer your third question, the quest for the best technique for inguinal hernia repair is a historic and continuous one. We do not have a single technique that seems to fit all. However, based on research conducted over the past decades, we do know that the preperitoneal position of the mesh has several advantages, and therefore, it is not surprising that, for example, in my country, 20% of the hospitals prefer an open preperitoneal repair method to repair a primary inguinal hernia. Besides these comments, it is worth mentioning that open preperitoneal techniques are particularly suitable for patients who cannot undergo general anesthesia, or even in the case of low- to middle-income countries.

### Frederik Berrevoet (Ghent, Belgium)

Congratulations on this nice study. How do you explain that the outcome of the transinguinal approach, with possible nerve damage, did not lead to clinical differences between the 2 groups?

### Response From Willem J.V. Bökkerink (Nijmegen, The Netherlands)

Of course, we would expect more chronic pain, due to the transinguinal approach; however, we do not see this in the present trial. We actually observed quite low rates of chronic pain, and this also shines another light on the problem of chronic pain. As we know already, it is multifactorial, and perhaps, pure inguinal nerve damage is not the only, or simply, a minor factor in the problem of chronic pain.

### Olivier Farges (Clichy, France)

Just as everyone tends to perform better when they do something that they love, the outcome of an operation might likewise be influenced by the pleasure that a surgeon has in performing it. Do you know which of the procedures is more enjoyed by surgeons? Don’t you think that the surgeon's enjoyment should also be one of the endpoints or a confounder in the evaluation of such studies?

### Response From Willem J.V. Bökkerink (Nijmegen, The Netherlands)

This also touches on the point about the learning curve. Let me say that the transinguinal approach is usually the one that most surgeons already know from the Lichtenstein technique, which most surgeons are still taught. A direct open preperitoneal technique is somewhat less familiar. It, however, gives you a beautiful, open view of the posterior plane. Both techniques are loved by those who do them, and it also slightly depends on your previous skills, regarding Lichtenstein, or maybe, your endoscopic skills.

I think that this can be a confounder, but should not be an endpoint, since I always prefer to adopt a patient-first mentality, especially when talking about an inguinal hernia. It's an operation that patients usually consider lightly, and it often goes well, without any complications. However, when complications arise, especially in terms of chronic pain, this can be a terrible course. So, please, let us put the patients first, followed by other factors, including the surgeon who performs the surgery.
