# Supplementary material for: Open Preperitoneal Inguinal Hernia Repair, TREPP Versus TIPP in a Randomized Clinical Trial
Source: Ann Surg. 2021 Aug 2;274(5):698–704. doi: 10.1097/SLA.0000000000005130 (PMC8500364; doi:10.1097/SLA.0000000000005130)

**SUPPLEMENTAL DIGITAL CONTENT**

Three files are submitted as Supplemental Digital Content (SDC)

1. SDC 1 The SF36 results.pdf

2. SDC 2 The CONSORT 2010 Checklist.doc

3. SDC 3 The CONSORT 2010 Flow diagram.doc

The SDC 1 The SF36 results.pdf file shows the mixed model analyses of the eight SF-36 domains comparing TREPP with TIPP patients at baseline, 2 weeks, 6 months and 1 year postoperatively.

The single file contains the following figures:

Figure SDC 1. Physical functioning domain SF-36 TREPP versus TIPP patients

Figure SDC 2. Role-Physical domain SF-36 TREPP versus TIPP patients

Figure SDC 3. Bodily Pain domain SF-36 TREPP versus TIPP patients

Figure SDC 4. General Health perception domain SF-36 TREPP versus TIPP patients

Figure SDC 5. Vitality domain SF-36 TREPP versus TIPP patients

Figure SDC 6. Social functioning domain SF-36 TREPP versus TIPP patients

Figure SDC 7. Role-emotional domain SF-36 TREPP versus TIPP patients

Figure SDC 8. Mental Health domain SF-36 TREPP versus TIPP patients

The SDC 2 The CONSORT 2010 Checklist.doc contains the filled out checklist from http://www.consort-statement.org/

The SDC 3 The CONSORT 2010 Flow diagram.doc contains the filled out Flow diagram from http://www.consort-statement.org/


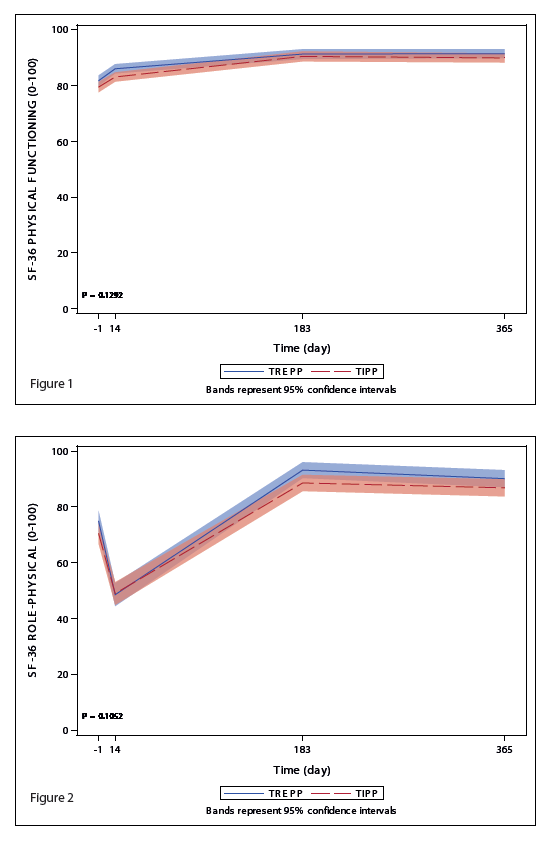


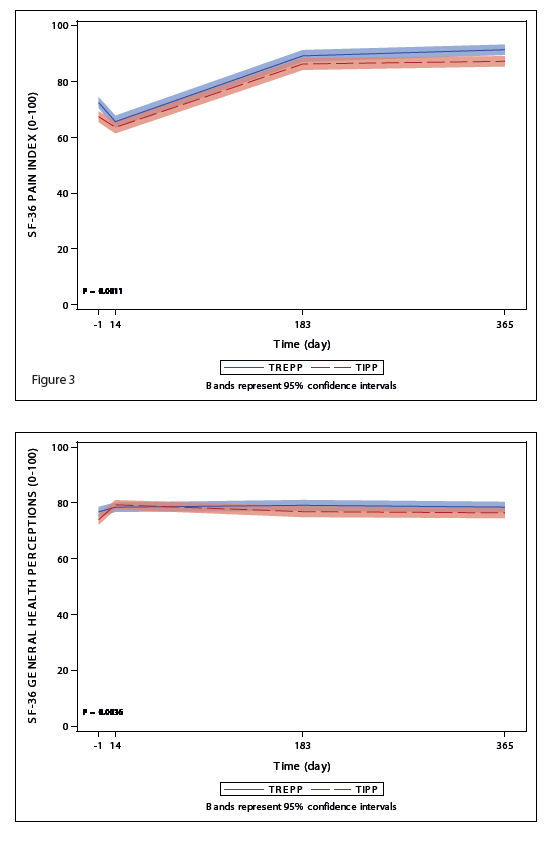


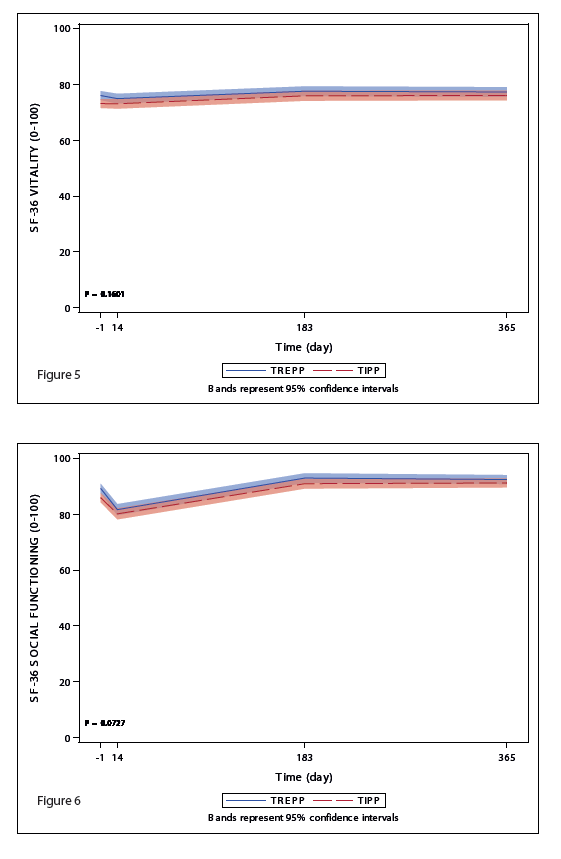


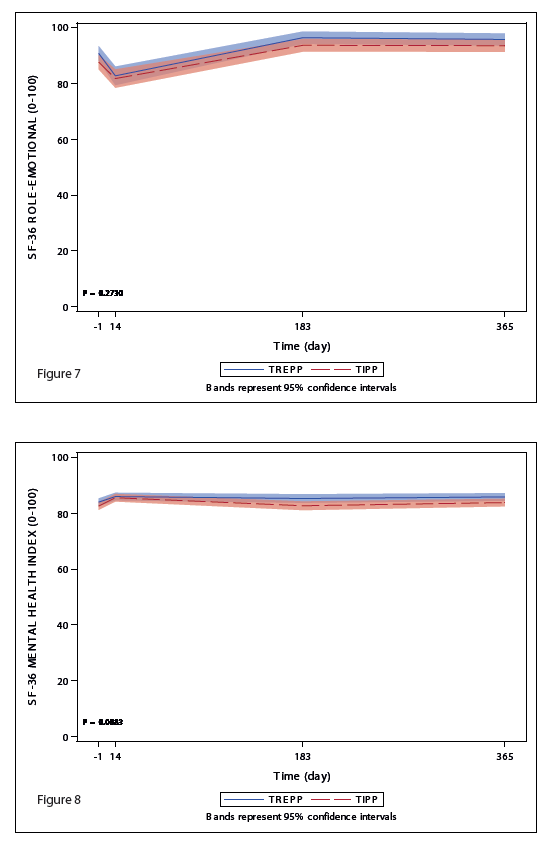

Supplement: Supplemental Digital Content [file ansu-274-0698-s001.doc]
